# Identification and validation of the angiogenic genes for constructing diagnostic, prognostic, and recurrence models for hepatocellular carcinoma

**DOI:** 10.18632/aging.103107

**Published:** 2020-05-06

**Authors:** Jinyu Zhu, Bufu Tang, Jie Li, Yueli Shi, Minjiang Chen, Xiuling Lv, Miaomiao Meng, Qiaoyou Weng, Nannan Zhang, Kai Fan, Min Xu, Jiansong Ji

**Affiliations:** 1 Key Laboratory of Imaging Diagnosis and Minimally Invasive Intervention Research, Lishui Hospital, School of Medicine, Zhejiang University, Lishui 323000, China; 2 Department of Radiology, Second Affiliated Hospital, School of Medicine, Zhejiang University, Hangzhou 310058, China; 3 Department of Pathology and Pathophysiology, School of Medicine, Zhejiang University, Hangzhou 310058, China; 4 Department of Radiology, The Fifth Affiliated Hospital of Wenzhou Medical University, Lishui 323000, China

**Keywords:** angiogenesis, hepatocellular carcinoma (HCC), diagnosis, recurrence, prognosis

## Abstract

Since angiogenesis has an indispensable effect in the development and progression of tumors, in this study we aimed to identify angiogenic genes closely associated with prognosis of HCC to establish diagnostic, prognostic, and recurrence models. We analyzed 132 angiogenic genes and HCC-related RNA sequence data from the TCGA and ICGC databases by Cox and least absolute shrinkage and selection operator (LASSO) regression, and identified four angiogenic genes (ENFA3, EGF, MMP3 and AURKB) to establish prognosis, recurrence and diagnostic models and corresponding nomograms. The prognostic and recurrence models were determined to be independent predictors of prognosis and recurrence (P < 0.05). And compared with the low-risk group, patients in the high-risk group had worse overall survival (OS) rates in training cohort (P < 0.001) and validation cohort (P < 0.001), and higher recurrence rates in training cohort (P<0.001) and validation cohort (P=0.01). The diagnostic models have been validated to correctly distinguish HCC from normal samples and proliferative nodule samples. Through pharmacological analysis we identified piperlongumine as a drug for targeting angiogenesis, and it was validated to inhibit HCC cell proliferation and angiogenesis via the EGF/EGFR axis.

## INTRODUCTION

Liver cancer is estimated to be the sixth most common cancer in the world (841,000 cases/year) and the fourth leading cause of cancer (782,000 cases/year), and it poses a serious health threat and economic burden to the world [1]. Hepatocellular carcinoma (HCC) accounts for the vast majority (approximately 75%-85%) of primary liver cancer, and its increasing worldwide incidence and mortality has generated major clinical interest. There are various treatment strategies for different characteristics of HCC. Surgical resection, transplantation, ablation, and trans-arterial chemoembolization have been shown to produce survival benefits [2, 3], but due to the high recurrence rate after surgery, the prognosis of HCC patients is still unsatisfactory, and OS and RFS are still very poor [4, 5]. Accurate prognosis assessment is a key step in the effective management and treatment of patients with HCC. In addition to traditional clinical variable, such as age, gender, pathological grade, clinical stage and vascular tumor cell invasion that affect the prognosis of HCC patients, complex molecular pathogenesis is also significantly related to the carcinogenicity and clinical outcome of HCC [6]. To develop more effective treatment management strategies for improving the survival status and clinical outcomes of HCC patients, it is urgent that new, efficient molecular prognostic markers be explored.

Angiogenesis is defined as the process of producing new blood vessels in existing vasculature in a manner known as "germination" [7]. Angiogenesis can occur in physiological and pathological conditions. Physiological angiogenesis is involved in embryonic development, wound healing, and collateral formation to improve organ perfusion, and it is essential in normal human development [8]. Pathological angiogenesis is one of the hallmarks of cancer [9], and it plays a crucial role from the early stages of tissue carcinogenesis to the end of tumor metastasis [10]. Angiogenesis in tumors is driven the overexpression of angiogenic factors and rarely matures or becomes resolved [11]; thus, it is the basis for tumor cell growth, invasion, and metastasis [12]. On the one hand, these new blood vessels provide the necessary oxygen and nutrients to maintain the rapid growth and proliferation of tumor cells, and on the other hand, they provide a channel for tumor cells to enter the circulation and develop distant metastasis [13].

HCC is one of the most common highly vascularized solid tumors [14], and angiogenesis plays a key role in the pathogenesis, growth and progression of HCC [15]. When precancerous lesions develop into clinical HCC, angiogenesis starts, and it continues with the progression of the tumor, inducing rapid growth, proliferation and metastasis of the tumor and causing poor prognosis of HCC patients [16]. Many previous studies have explored and reported the close relationship between angiogenesis and HCC. For example, Park YN [17] analyzed vascular endothelial growth factor (VEGF), which is the most important factor in tumor angiogenesis. Its expression level in tumor tissues increases with the progression of HCC. VEGF-positive HCC patients have a worse prognosis and shorter survival time than their VEGF-negative counterparts. Llovet JM et al. [18] also confirmed that angiogenic markers such as plasma angiopoietin 2 (Ang2) and plasma VEGF are independent predictors of survival prognosis in patients with advanced HCC. However, most of the current research is focused on exploring the effects of individual angiogenesis-related genes on the development and prognosis of HCC. Few studies have integrated multiple angiogenesis-related genes by high-throughput biomarker sequencing and comprehensively analyzed the relationship between these genes and the prognosis and survival of HCC.

In this study, by analyzing the HCC-related mRNA sequence data in TCGA database and the ICGC database and the angiogenic genes identified in previous publications, we identified four angiogenic genes significantly related to the prognosis of HCC and then constructed and validated diagnostic, prognostic and recurrence models and corresponding nomograms of HCC. The possible anti-tumor mechanisms of piperlongumine, an inhibitor of angiogenic genes, were comprehensively evaluated. Our findings may help further improve the individualized treatment and clinical outcome of HCC.

## RESULTS

### Differentially expressed HCC-related angiogenic gene identification

**Figure 1:**
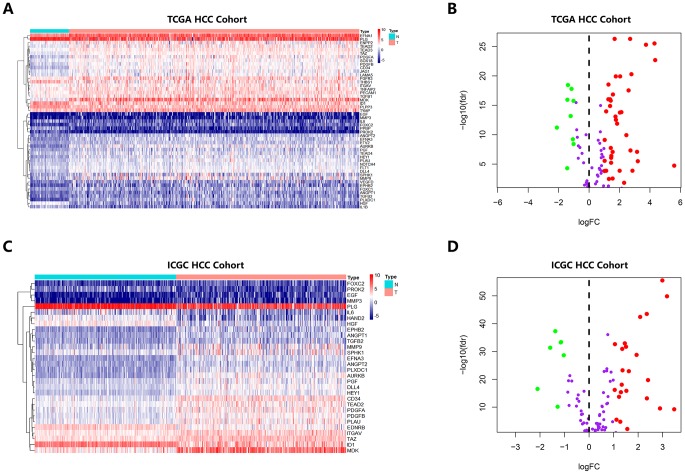
**Heatmap and Volcano Plot showed the differentially expressed angiogenic genes of HCC patients in different database.** (**A**, **B**) Gene expression features in the TCGA database. (**C**, **D**) Gene expression levels in the ICGC database.

First, there is a flowchart that graphically illustrates the analysis process for this study Supplementary Figure 1. We identified 132 angiogenic genes from several studies on angiogenesis, and we matched these genes to HCC-related mRNA sequence data in TCGA and ICGC databases; we compared differentially expressed genes (DEGs) in the mRNA expression profiles between HCC tissues and adjacent noncancerous tissues. Using the threshold of FDR < 0.05 and |log2 FC| > 1, 49 differentially expressed angiogenic genes (40 upregulated and 9 downregulated) were obtained from the database from TCGA (Figure 1A, 1B), and 30 differentially expressed angiogenic genes (24 upregulated and 6 downregulated) were obtained in the ICGC database (Figure 1C, 1D). Then, by matching the angiogenic genes in the two databases, we selected 28 genes for subsequent analysis (23 upregulated and 5 downregulated) (Supplementary Figure 2).

### Identification of key angiogenic genes closely associated with prognosis of HCC

To identify angiogenic genes that were significantly associated with HCC survival, we performed LASSO regression and Cox regression analysis. We first performed LASSO to reduce the number of angiogenic genes. A total of 1000 Cox LASSO regression iterations and 10 cross-validations were performed using the R package glmnet. The higher the nonzero coefficient appeared after 1000 iterations of Cox LASSO regression, the stronger the ability of the corresponding gene to predict prognosis. Using the Cox LASSO regression, we selected 14 angiogenic genes with a strong predictive prognosis (with a recurrence frequency greater than 900 times out of 1000 repetitions) (Supplementary Figure 3). We then used 370 HCC samples from the database from TCGA along with OS time and survival status to investigate the relationship between these 14 angiogenic genes and prognosis using univariate Cox regression analysis. The results confirmed that there were 4 genes (EFNA3, EGF, MMP3 and AURKB) with statistical significance (P<0.01); that is, these four key angiogenic genes were significantly associated with HCC prognosis.

### Construction of an angiogenesis-related prognostic signature and validation of its predictive performance

**Figure 2:**
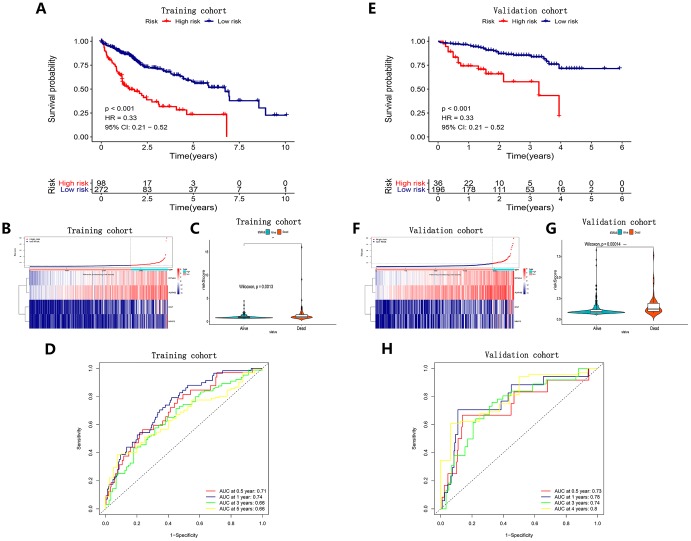
**Survival analysis, risk score distribution, Violin plots and time-dependent ROC curves of a prognostic model in the TCGA HCC cohort** (**A**–**D**) and ICGC HCC cohort (**E**–**H**). A and E K-M survival curves showed the OS in the high-risk group was significantly worse than that in the low-risk group (P<0.05). (**B**, **F**) Distribution of risk scores of HCC patients with different gene expression levels. (**C**, **G**) Risk scores of HCC patients with different survival status. (**D**, **H**) ROC curve analysis for OS prediction.

Based on these four angiogenic genes, we established a prognostic signature by multivariate Cox regression as follows: prognostic index (PI) = (β* expression level of EGF) + (β* expression level of MMP3) + (β* expression level of AURKB) + (β* expression level of EFNA3). In the HCC cohort from TCGA, 371 HCC patients with survival status were divided into high-risk and low-risk groups using the optimal cut-off value determined by X-tile software. The OS of the high-risk group was significantly worse than that of the low-risk group (P < 0.001) (Figure 2A). Figure 2B shows the risk score distribution corresponding to the expression level of the genes. In HCC patients with different survival statuses, we found that the risk score of the dead patients was significantly higher than that of the living patients (P<0.01) (Figure 2C). Time-dependent ROC curve analysis was performed to evaluate the specificity and sensitivity of the prognostic signature. The higher the area under the curve (AUC), the better the predictive performance of the prognostic signature. The AUCs of the prognostic signature reached 0.76 at 0.5 years, 0.74 at 1 year, 0.67 at 3 years and 0.65 at 5 years, indicating a great predictive value of the prognostic signature for OS (Figure 2D). For further validation of the prediction ability, the prognostic signature was applied to the ICGC database (containing 243 HCC samples) for survival prediction. Utilizing the formula mentioned earlier and the optimal cutoff value identified by X-tile software, 243 HCC patients were classified into high-risk and low-risk groups, and consistent with previous results, patients in the high-risk group had a significantly worse OS than those in the low-risk group (P < 0.001) (Figure 2E). Figure 2F shows the risk score distribution corresponding to the expression level of the genes. Consistent with previous findings, the risk score of dead HCC patients was much higher than that of living HCC patients (P<0.01) (Figure 2G). The AUCs of the prognostic signature for OS were 0.73 at 0.5 years, 0.79 at 1 year, 0.64 at 3 years and 0.70 at 4 years (Figure 2H).

### A predictive nomogram for OS prediction was constructed and validated in the HCC cohort from TCGA

**Figure 3:**
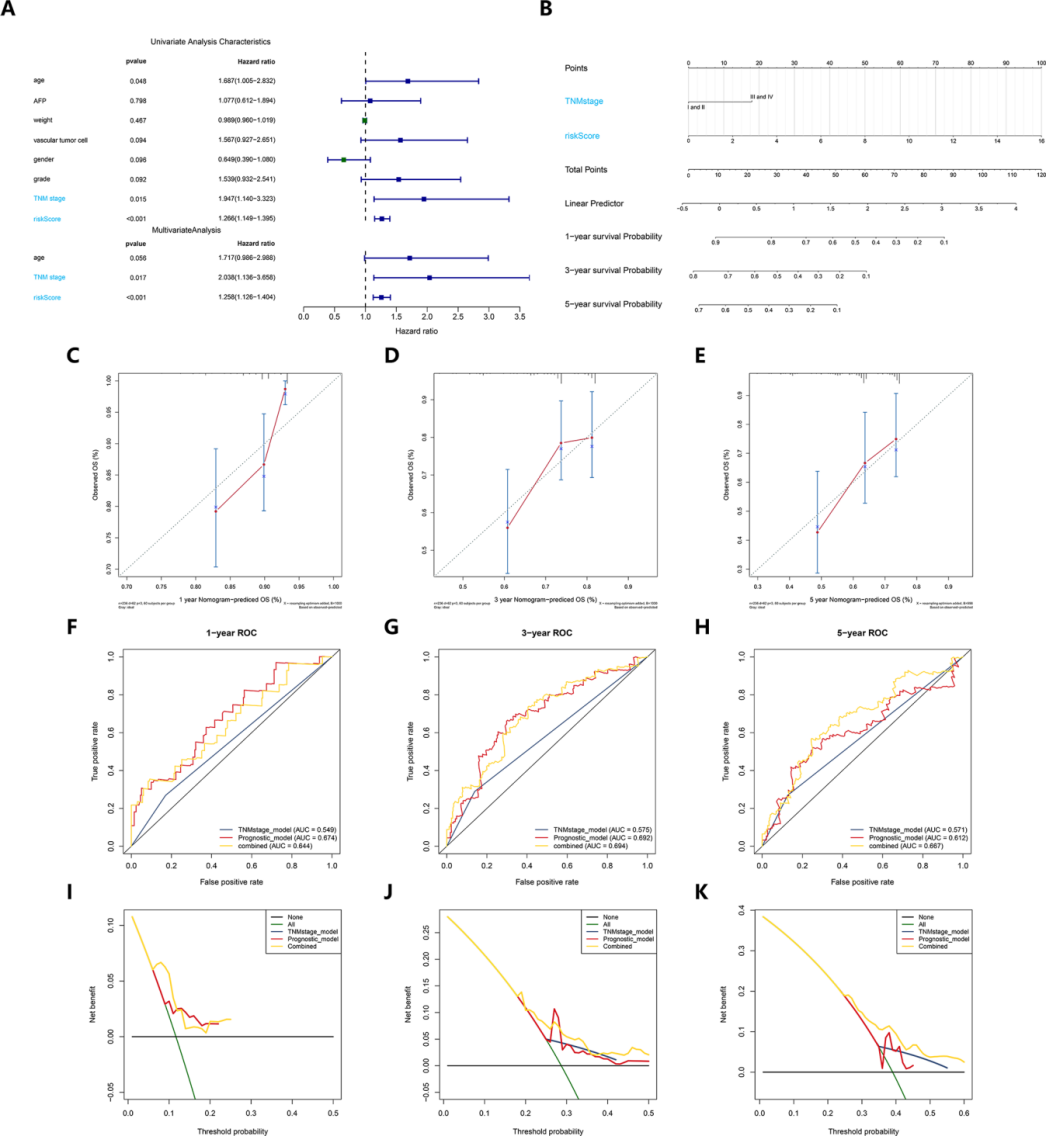
**Construction and verification of a predictive nomogram.** (**A**) Univariate and multivariate Cox regression suggested the prognostic model and age were independent prognostic predictors. (**B**) The nomogram for OS prediction in HCC patients at 1, 3, and 5 years. (**C**–**E**) Calibration curves of nomogram for OS prediction at 1, 3, and 5 years. (**F**–**H**) ROC curves to evaluate the predictive performance of nomogram. (**I**–**K**) DCA curves to evaluate the clinical decision-making benefits of nomogram.

To evaluate the independent predictive value of this prognostic signature, we performed univariate and multivariate Cox regression analyses on the risk score of the prognostic signature and clinical characteristics (AFP, weight, vascular tumor invasion, sex, pathological grade, and TNM stage) of 370 HCC patients in the cohort from TCGA. The results of the univariable Cox regression analysis showed that age, TNM stage and the risk score of the prognosis signature had predictive value for OS (HR>1.00, P < 0.05), while AFP, weight, vascular tumor invasion and sex were not associated with OS. Therefore, we incorporated age, TNM stage, and risk score of the prognostic signature in a multivariate Cox regression analysis, and the results showed that TNM stage (HR = 1.961, P < 0.05) and the prognostic signature (HR = 1.315, P < 0.05) were important independent prognostic factors for OS (Figure 3A). To provide clinicians with a quantitative method to more accurately predict the prognosis and survival status of HCC patients, thereby contributing to more individual clinical decisions and treatment options, we constructed a predictive nomogram, which integrated the two independent prognosis factors based on the multivariable Cox regression analysis (Figure 3B). Each independent prognosis factor was assigned a certain score scale, and the total point value was obtained by adding up the specific points corresponding to the individual's prognosis variable, thereby determining the corresponding OS for the individual at 1, 3, and 5 years. The calibration curves of the nomogram at 1, 3, and 5 years were very close to the best prediction curve, showing a great consistency between the predicted OS rates and the actual observations (Figure 3C–3E).

We then adopted the C-index, time-dependent ROC and DCA to verify the prognostic accuracy of the nomogram in OS prediction. The C index of the nomogram in OS prediction was 0.64, with 1000 cycles of bootstrapping (95% CI: 0.56-0.72), which was superior to the C index for the TNM stage (0.54) and the prognostic signature (0.63); these results were consistent between the nomogram’s prediction and actual results. The time-dependent ROC curves showed that the nomogram with the integration of two independent prognosis factors reached 0.641, 0.681 and 0.671 in 1 year, 3 years and 5 years, which was better than a single independent prognosis factor on predictive performance (Figure 3F–3H). The results of the DCA also showed that compared to a single independent prognostic factor, the best net benefit was obtained by prognosis prediction with the nomogram (Figure 3I–3K).

### Definition of a recurrence signature based on the key angiogenic genes

**Figure 4:**
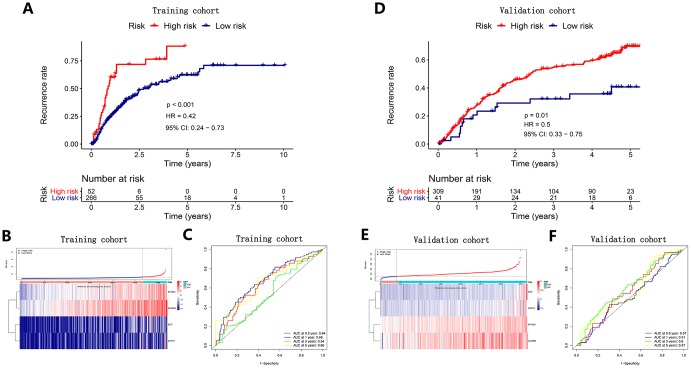
**Survival analysis, risk score distribution and time-dependent ROC curves of a recurrence model in the TCGA HCC cohort** (**A**–**C**) and ICGC HCC cohort (**D**–**F**). (**A**, **D**) The recurrence rates in the high-risk group was much higher than that in the low-risk group (P<0.05). (**B**) and E Gene expression levels and corresponding risk scores in HCC patients. (**C**, **F**) ROC curve analysis for recurrence prediction.

We utilized the regression coefficient (β’) of a multivariate Cox proportional hazards model based on four angiogenic genes and patients with release-free survival (RFS) time and recurrent status in the cohort from TCGA and the GSE14520 cohort. Then, we further constructed a recurrence signature as follows: prognostic index (PI) = (β’* expression level of EGF) + (β’* expression level of MMP3) + (β’* expression level of AURKB) + (β’* expression level of EFNA3). Determining the optimal cut-off value by X-tile software, HCC patients were divided into high-risk and low-risk groups. We found that the recurrence rate in HCC patients in the high-risk group was dramatically higher than that of the low-risk group in the same period in the cohort from TCGA (P<0.001) (Figure 4A) and the GSE14520 cohort (P=0.01) (Figure 4D). The distribution of risk scores corresponding to the expression patterns of the angiogenic genes is presented in Figure 4B, 4E. The AUC values for the 0.5-, 1-, 3-, and 5-year recurrence probability with the recurrence signature reached 0.64, 0.68, 0.54 and 0.66 in the cohort from TCGA, respectively (Figure 4C). In addition, the AUCs for 0.5-, 1-, 3-, and 5-year recurrence probability with the recurrence signature reached 0. 57, 0.51, 0.6 and 0.57 in the GSE14520 cohort, respectively (Figure 4F). These results showed that the recurrence signature had a great predictive power for recurrence.

### Establishment and evaluation of a recurrence nomogram in predicting recurrent probability in the HCC cohort from TCGA

**Figure 5:**
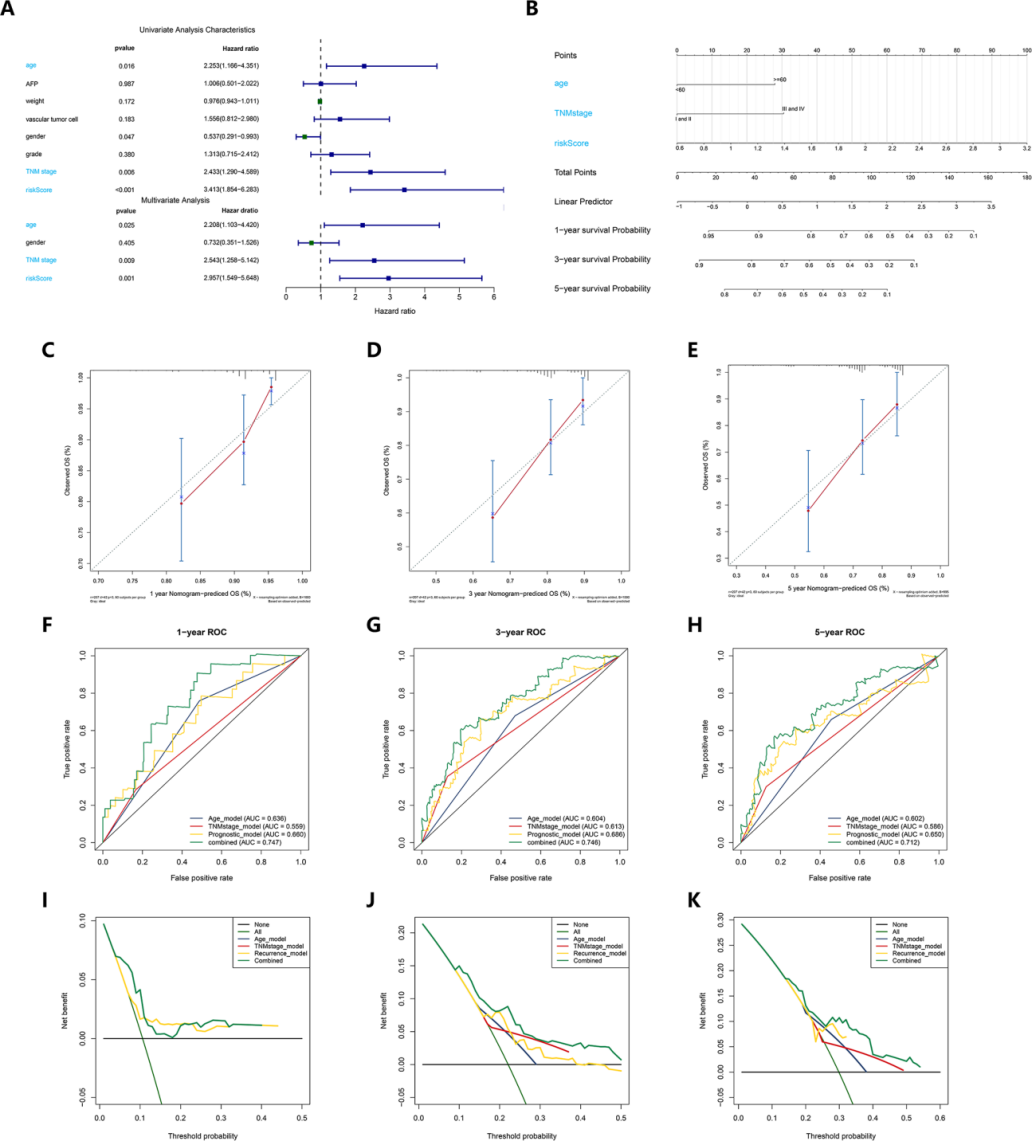
**Construction and validation of a recurrence nomogram.** (**A**) Univariate and multivariate Cox regression suggested the recurrence model, TNM stage and age were independent prognostic predictors. (**B**) The nomogram for recurrence prediction in HCC patients at 1, 3, and 5 years. (**C**–**E**) Calibration curves of nomogram for recurrence prediction at 1, 3, and 5 years. (**F**–**H**) ROC curves to evaluate the predictive performance of nomogram. (**I**–**K**) DCA curves to evaluate the clinical decision-making benefits of nomogram.

To determine whether the predictive performance of the recurrence signature was independent of traditional clinical features, we performed univariate and multivariate Cox regression analysis on these variables in the cohort from TCGA. The analyses confirmed that age (HR =2.198, P < 0.05), TNM stage (HR = 2.499, P < 0.05) and the recurrence signature (HR = 2.883, P < 0.05) were independent of other clinical features (Figure 5A). Then, a recurrence nomogram integrated the three significantly independent factors was established (Figure 5B). The calibration curves of the nomogram for 1-, 3-, and 5-year recurrence probabilities, and the best prediction curve (45°line) was nearly coincident, indicating a great agreement between the predicted recurrence probabilities and the actual observations (Figure 5C–5E).

Meanwhile, we compared the prediction accuracy between the nomogram and other single individual prognostic factors via C index, time-dependent ROC curve analysis and DCA. The C index of the nomogram reached 0.71, indicating that the nomogram had an excellent predictive accuracy for recurrence in HCC patients. The AUCs for 1-, 3-, and 5-year recurrence probability with the nomogram reached 0.749, 0.748, 0.54 and 0.722, respectively (Figure 5F–5H). The DCA also demonstrated that the nomogram could help clinicians obtain the best net benefits when making clinical decisions (Figure 5I–5K). It was demonstrated that the nomogram had superior predictive performance and clinical value to that of any single independent recurrence factor.

### Identification and validation of a diagnostic model based on the four angiogenic genes

**Figure 6:**
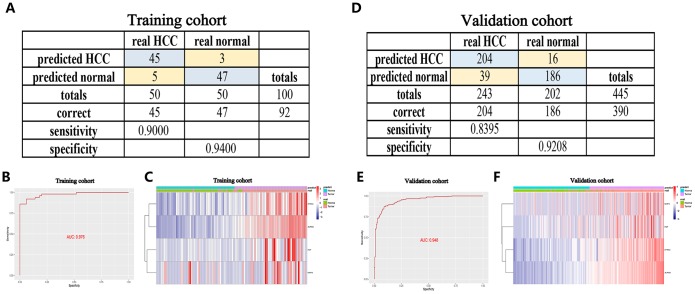
**A diagnostic model for distinguishing HCC from normal samples in the TCGA HCC cohort** (**A**–**C**) and ICGC HCC cohort (**D**–**F**). (**A**, **D**) Confusion matrix for the binary classification results of the diagnostic model. (**B**, **E**) ROC curves for the predictive performance evaluation of the diagnostic model. (**C**, **F**) Unsupervised hierarchical clustering of the four angiogenic genes for the diagnostic model.

To improve the disease outcome of HCC patients, there is a need for prognostic signatures and recurrence signatures to help clinicians adjust the treatment strategy for confirmed HCC patients, but in the early diagnosis, there is also a need for sensitive and specific diagnostic criteria to accurately distinguish HCC patients from normal subjects. To this end, we performed a stepwise logistic regression method to establish a diagnostic model that integrated these four angiogenic genes to distinguish between HCC patients and normal subjects. The diagnostic equation was finally identified as follows: logit (P = HCC) = -75.2125977 + (0.85 × ENFA3 expression level) + (2.61 × EGF expression level) + (3.92 × MMP3 expression level) + (9.98 × AURKB expression level). The diagnostic model was confirmed to achieve a specificity of 94.00% and a sensitivity of 90.00% in the HCC cohort from TCGA (containing 50 normal samples and paired 50 HCC samples) (Figure 6A). The AUC for the diagnostic model reached 0.976, which is a diagnostic value that is superior to other methods (Figure 6B). We then validated the diagnostic model using the ICGC HCC cohort (containing 202 normal samples and 243 HCC samples). The diagnostic model diagnosed HCC patients with a specificity of 92.08%, sensitivity of 83.95% (Figure 6D), and AUC of 0.948 (Figure 6E), confirming that the diagnostic model can accurately distinguish HCC samples from normal samples. We show the correspondence between the predicted results of the diagnostic model and the actual status and the corresponding expression characteristics of angiogenic genes in Figure 6C, 6F.

**Figure 7:**
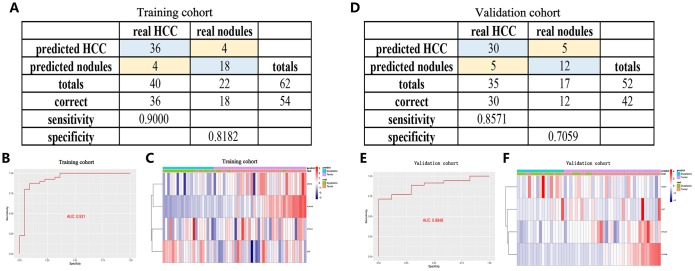
**A diagnostic model for distinguishing HCC from proliferative nodules in the training dataset (GSE89377) and validation dataset (GSE6764).** (**A**, **D**) Confusion matrix for the binary classification results of the diagnostic model. (**B**, **E**) ROC curves for the predictive performance evaluation of the diagnostic model. (**C**, **F**) Unsupervised hierarchical clustering of the four angiogenic genes for the diagnostic model.

Furthermore, to improve the early screening rate of HCC and reduce the probability of misdiagnosis, we integrated these four angiogenic genes to establish a diagnostic model in the GSE89377 dataset (containing 22 proliferative nodules samples and 40 HCC samples) to detect whether the diagnostic model could correctly distinguish between HCC and proliferative nodules. We used a stepwise logistic regression method to establish the diagnostic model with a diagnostic formula of logit (P = HCC) = -5.58 + (5.84 × ENFA3 expression level) + (1.84 × EGF expression level) + (-1.04 × MMP3 expression level) + (18.10 × AURKB expression level). The diagnostic model was confirmed to distinguish between HCC and proliferative nodules with high specificity (81.82%) and high sensitivity (90.00%) (Figure 7A). The AUC for the diagnostic model was 0.931, confirming that the diagnosis model had diagnostic capabilities superior to those of other methods (Figure 7B). We then applied this diagnostic model to GSE6764 to further validate its diagnostic performance. The results showed that the diagnostic model had a specificity of 70.59%, a sensitivity of 85.71% (Figure 7D), and an AUC of 0.8848 (Figure 7E), demonstrating that the diagnostic model correctly distinguished HCC and hyperplastic nodules. Figures 7C, 7F show different angiogenic gene expression levels, the correspondence between the predicted results in the diagnostic model and the actual results.

### Further internal and external validation of the expression patterns and prognostic predictive value of the angiogenic genes

**Figure 8:**
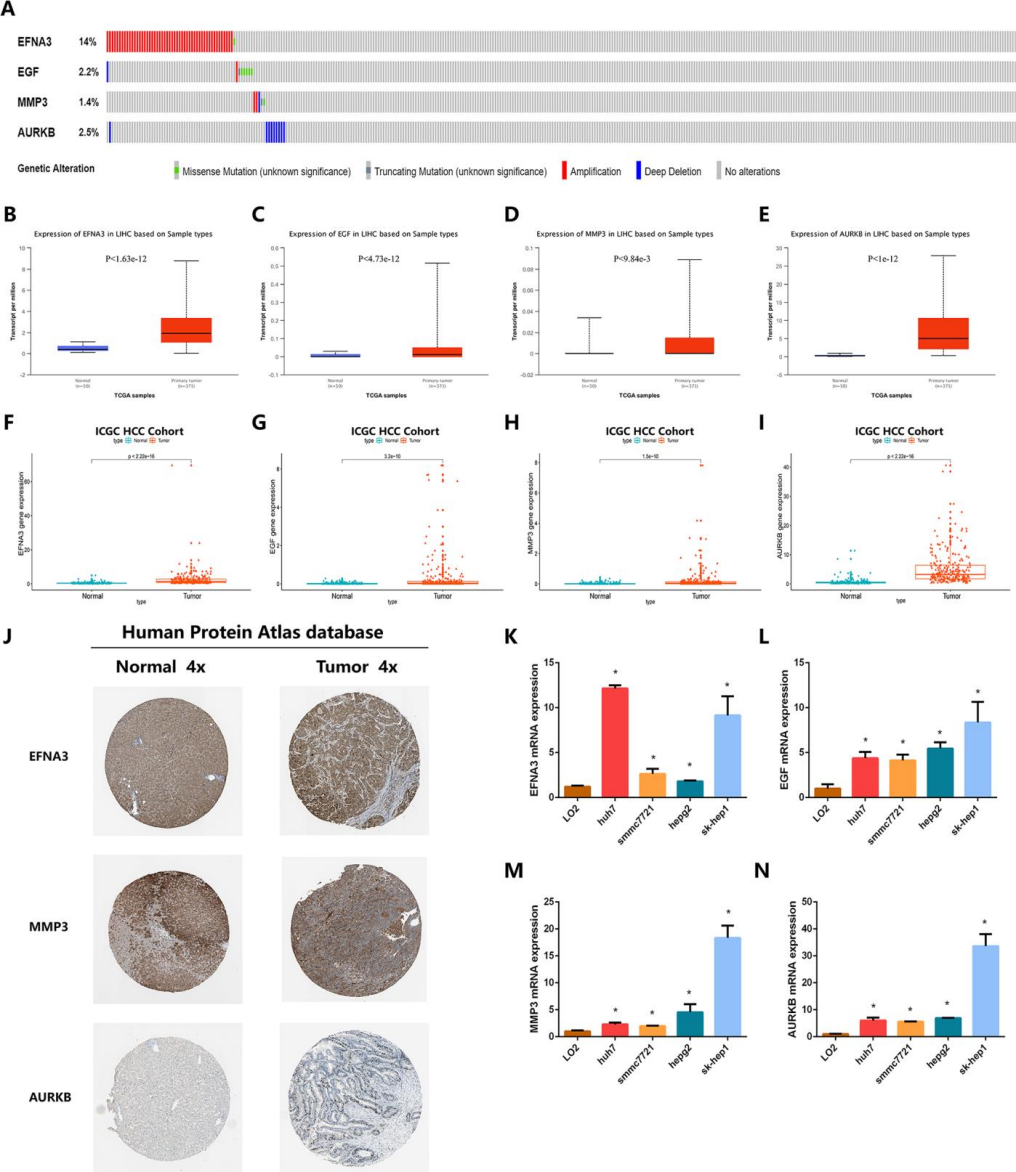
**Validation of the expression characteristics of angiogenic genes.** (**A**) Genetic alteration detection of the angiogenic genes in the TCGA HCC cohort. (**B**–**E**) Expression levels of angiogenic genes in HCC and normal samples in the TCGA HCC cohort. (**F**–**I**) Expression levels of angiogenic genes in HCC and normal samples in the TCGA HCC cohort. (**J**) The imagines showed the expression patterns of the angiogenic genes in HCC tissues and normal liver tissues. (**K**–**N**) The expression profiles of the angiogenic genes in multiple types of HCC cell lines.

To examine the value of these four angiogenic genes (ENFA3, EGF, MMP3, and AURKB) for the construction of diagnostic, prognostic and recurrence models, we analyzed the expression patterns of the angiogenic genes in the HCC cohorts from ICGC database and different online database, including cBioportal, UALCAN and Human Protein Atlas database. We first performed genetic alteration detection in the HCC cohort from cBioportal database [19]. It was found that 20.1% (72 HCC samples) showed genetic alterations, 14% of which occurred in ENFA3, where the main alteration was amplification; 5 mutations occurred in EGF (2.2%) (Figure 8A). These genes have the potential to become targeted therapeutic sites and provide new therapeutic targets for the treatment of HCC. Subsequently, in the HCC cohort from UALCAN database [20], we found that the expression levels of the four angiogenic genes in tumor tissues were significantly higher than it was in normal tissues (P < 0.001) (Figure 8B–8E), and this finding was also consistently validated in the ICGC HCC cohort (P < 0.001) (Figure 8F–8I). In addition, to verify the clinical relevance of these four angiogenic genes, we analyzed the expression of proteins encoded by the four genes in clinical HCC specimens and normal specimens from the Human Protein Atlas database [21]. The results showed that ENFA3, MMP3 and AURKB were strongly positive in HCC tissues compared to normal liver tissues (Figure 8J), while EGF was not found in the database. The expression characteristics of angiogenic genes were consistent with the prognosis and recurrence analysis, and the finding indicated that the expression characteristics of these four genes are of great value for constructing diagnostic, prognostic and recurrent models. In addition, the expression levels of the four genes in different cell lines are shown in Figure 8K–8N.

**Figure 9:**
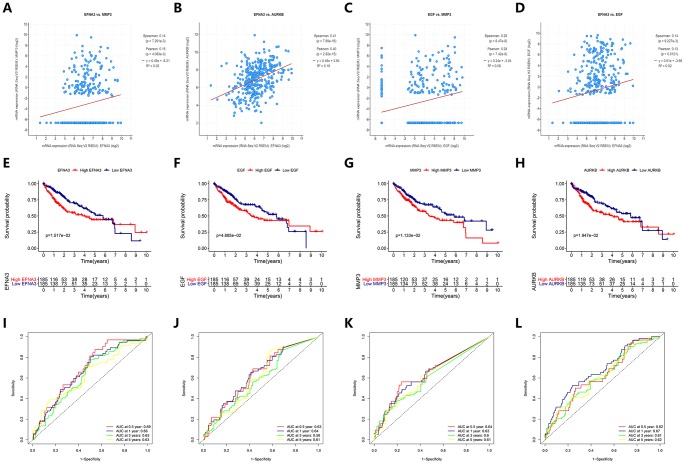
**Prediction performance of angiogenic genes for OS and regression analysis between expression levels of different genes.** (**A**–**D**) The expression of the angiogenic genes was synergistic with each other. (**E**–**H**) (**K**–**M**) survival curves indicated the OS in the high-expression group was significantly poorer than that in the low-expression group (P<0.05). (**I**–**L**) ROC curve analysis for OS prediction at 0.5, 1, 3 and 5 years.

Since these four angiogenic genes were highly expressed in HCC patients and were risk factors, we explored the expression correlation of these four genes. We found that ENFA3 and MMP3 have synergistic effects, and their expression levels were positively correlated (Pearson 0.15, P<0.05) (Figure 9A). This result was also verified among other genes (ENFA3 and AURKB, EGF and MMP3, EFNA3 and EGF) (P<0.05) (Figure 9B–9D), while the expression level of AURKB was not significantly correlated with the expression levels of EGF or MMP3 (P>0.05). These results suggested that there was a relationship between these genes and their expression, further contributing to the occurrence of HCC. To verify the association between the expression levels of these four angiogenic genes and the OS of HCC patients, we performed K-M curve analysis of the HCC cohort from TCGA. The analysis showed that the OS of HCC patients with high expression of EFNA3 was significantly lower than that of patients with low expression of ENFA3 (P<0.05). EGF, MMP3 and AURKB also had consistent results; that is, high expression of these genes corresponded to low OS (Figure 9E–9H). The AUCs for 0.5-, 1-, 3- and 5-year OS indicated a great predictive performance based on the expression of the four genes (Figure 9I–9L).

### Piperlongumine was found to be an inhibitor of the angiogenic genes

**Figure 10:**
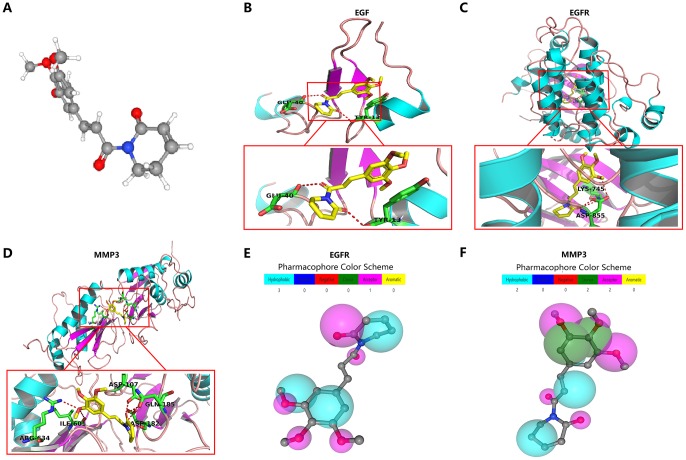
**Molecular docking and pharmacophore models for targeted drug identification.** (**A**) The 3D structure of piperlongumine was obtained from the PubChem database (https://pubchem.ncbi.nlm.nih.gov). (**B**–**D**) Specific binding site of piperlongumine and EGF (**B**), EGFR (**C**) and MMP3 (**D**). (**E**, **F**) The pharmacophore model of EGFR (**E**) and MMP3 (**F**).

In exploring the clinically targeted therapeutic value of the four angiogenic genes, we identified piperlongumine as an inhibitor of the EGF/EGFR axis through the Cancer Therapeutics Response Portal (CTRP) database (http://portals.broadinstitute.org/ctrp/). (Supplementary Figure 4). Piperlongumine is an amide alkaloid of *Piper longum* L. (long piper) that exhibits cytotoxicity against a variety of human cancer cell lines and exhibits antitumor activity in rodents [22]. The 3D structure of piperlongumine is shown in Figure 10A. To investigate the effect of piperlongumine on the angiogenesis signature, we first used Autodock software (Version 3.6.1) to dock piperlongumine and angiogenic genes, and we found that piperlongumine could effectively dock to specific sites of EGF, EGFR and MMP3 (Figure 10B–10D, Supplementary Figure 5). When further validation was performed using PharmMapper (http://www.lilab-ecust.cn/pharmmapper/), we found that EGFR and MMP3 were potential targeting regulators of piperlongumine (Figure 10E–10F).

### Analysis of the anti-tumor effect of piperlongumine and its possible molecular mechanism

**Figure 11:**
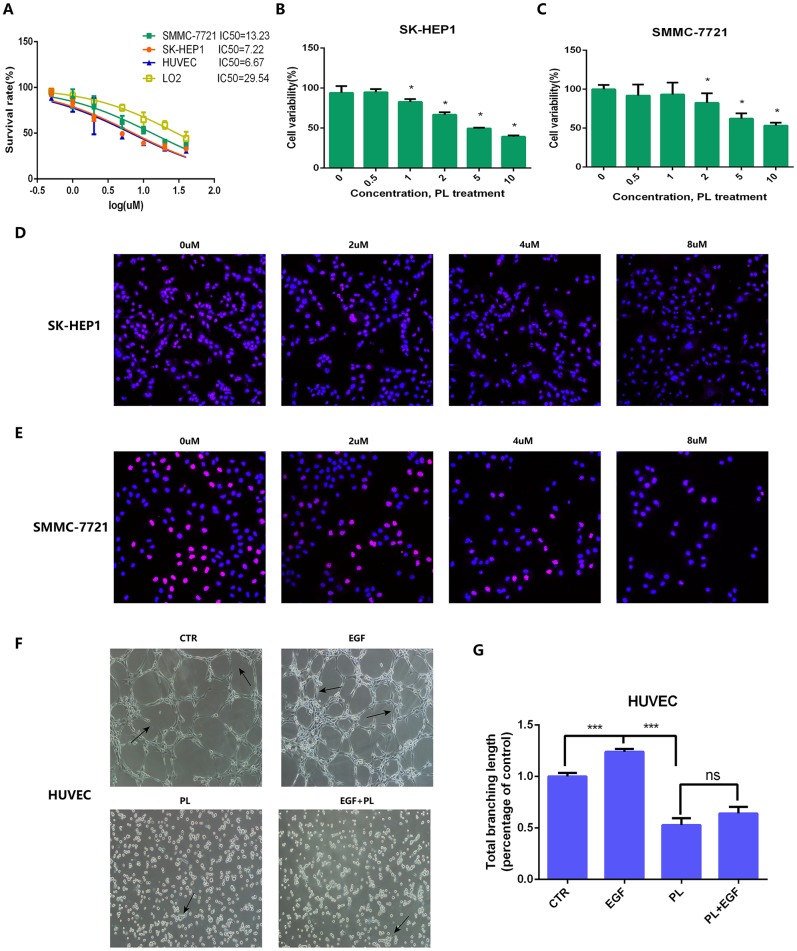
**IC50, CCK8 assay, EdU assay and tube formation assay for anti-tumor effect validation of piperlongumine.** (**A**) IC50 of piperlongumine in normal liver cell line (LO2), HUVEC and HCC cell lines. (**B**, **C**) CCK8 assay showed piperlongumine inhibited proliferation of SK-HEP1 (**B**) and SMMC-7721 (**C**) cell lines in a dose-dependent manner. (**D**, **E**) EdU assay showed the inhibition effect of piperlongumine in proliferation of SK-HEP1 (**D**) and SMMC-7721 (**E**) cell lines. (**F**) Tube formation assay suggested that piperlongumine inhibited angiogenesis via EGF/EGFR axis. (**G**) Statistical analysis to quantify the inhibitory effect of piperlongumine on angiogenesis.

To further verify whether piperlongumine had anti-tumor effects on HCC, we performed in vitro cell proliferation experiments and tube formation experiments. In the Half Maximal Inhibitory Concentration (IC50) assay, the IC50 of piperlongumine was found to be 7.22 μmol in the SK-HEP1 cell line, 13.23 μmol in the SMMC-7721 cell line, 6.67 μmol in the HUVEC cell line, and 29.54 μmol in human LO2 hepatocytes, indicating that HCC cell lines (SK-HEP1 and SMMC-7721) were much more sensitive than human LO2 hepatocytes to piperlongumine (Figure 11A). To assess the effect on tumor cell proliferation in vitro, we treated SK-HEP1 and SMMC-7721 cell lines with different concentrations of piperlongumine and evaluated them by CCK8 assay, and EdU assay. We found by the CCK8 assay that piperlongumine treatment inhibited cell proliferation in a dose-dependent manner (Figure 11B, 11C), and the EdU assay further confirmed this result (Figure 11D–11E). To investigate the effect of piperlongumine on angiogenesis in the EGF/EGFR signaling pathway, we performed an in vitro HUVEC tube formation assay. The results suggested that piperlongumine inhibited angiogenesis via the EGF/EGFR axis signaling pathway (Figure 11F–11G).

**Figure 12:**
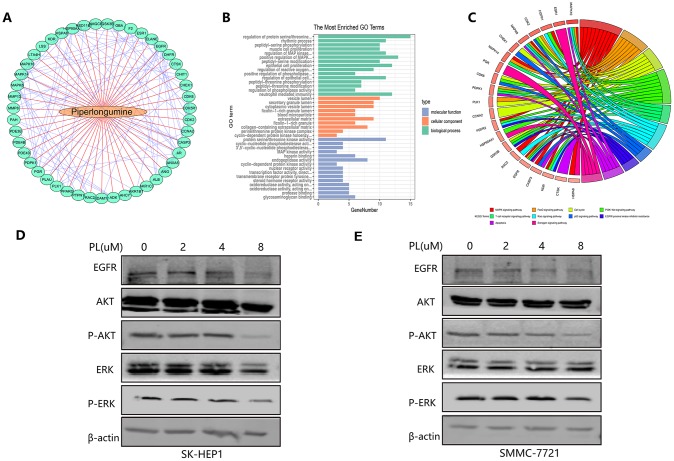
**Molecular mechanism of piperlongumine to achieve antitumor effect.** (**A**) The interaction of the corresponding encoded proteins of 226 angiogenic genes regulated by piperlongumine. (**B**–**C**) The GO analysis (**B**) and the KEGG pathway analysis (**C**) of the 226 angiogenic genes. (**D**–**E**) Western blot analysis of SK-HEP1 (**D**) and SMMC-7721 (**E**) cell lines.

Because we have demonstrated that the anti-tumor effect of piperlongumine was achieved by inhibiting proliferation and angiogenesis, we attempted to clarify the specific mechanism of piperlongumine on HCC cells. In the Cancer Therapeutics Response Portal (CTRP) database (http://portals.broadinstitute.org/ctrp/), we found that 226 genes could be regulated by piperlongumine, and the coexpression features of the corresponding encoded proteins are shown in Figure 12A. Gene Ontology (GO) and Kyoto Encyclopedia of Genes and Genomes (KEGG) pathway enrichment analyses were performed on these 226 genes. The results suggested that piperlongumine could cause changes in the EGFR tyrosine kinase inhibitor resistance pathway (P<0.01) (Figure 12B, 12C). To further validate the specific mechanism of piperlongumine, we treated SK-HEP1 and SMMC-7721 cells with different concentrations of piperlongumine and then performed western blot assays. As shown in Figure 12D, 12E, piperlongumine treatment reduced the levels of EGFR, p-Akt and p-Erk in a dose-dependent manner, suggesting that piperlongumine inhibited angiogenesis-induced tumorigenesis by targeting EGFR to influence the EGFR/EGF signaling pathway.

## DISCUSSION

With the high recurrence rate and low survival rate after surgery, liver cancer is known to be one of the most deadly malignant tumors worldwide [23]. Many methods have been used to evaluate the prognosis of patients with HCC, such as the American Joint Committee on Cancer (AJCC) staging system, the Barcelona Clinic Liver Cancer (BCLC) classification, and the Cancer of the Liver Italian Program (CLIP) staging system [24], but these assessment criteria were based on clinical features and did not consider the critical role of complex molecular pathogenesis in the carcinogenicity and disease outcome of HCC; further, the prognostic predictive performance of these assessment methods was also unsatisfactory. Previous studies have focused on exploring and discovering certain molecular markers and have found that their combinations have good predictive value in the prognosis and survival of HCC patients [25, 26], but molecular markers that have been identified that have good prognostic value are still rare.

Angiogenesis is required for tissue repair, remodeling in physiological processes such as reproduction, embryo development and wound healing, and it is a hallmark of disease states such as cancer, rheumatoid arthritis and atherosclerosis [27, 28]. As a highly angiogenic tumor, HCC growth and development depends on angiogenesis [29]. Angiogenesis in HCC tissue is primarily triggered by the release of overexpressed angiogenic factors from malignant cells and the tumor microenvironment [14]. The expression patterns of angiogenic factors are closely related to the poor prognosis of HCC patients and can be used as potential therapeutic targets [30]. However, the number of identified angiogenic factors that are associated with the prognosis of HCC patients is still low; further, most of the studies focus on the effects of individual genes on HCC disease outcomes, and few studies have combined several angiogenic factors to explore the prognosis of HCC.

The widespread use of high-throughput arrays provided an opportunity for us to seek new angiogenic genes involved in the carcinogenicity of HCC and to perform combinatorial analysis [31]. In this study, we identified angiogenic genes that were abnormally expressed in HCC by analyzing HCC-related mRNA sequences in TCGA and ICGC databases, and then we identified expression characteristics of four angiogenic genes (ENFA3, EGF, MMP3 and AURKB) that were significantly associated with HCC prognosis using univariate Cox regression and LASSO Cox regression. Prognostic models, recurrence models, and corresponding nomograms of these four angiogenic genes were constructed by multivariate Cox proportional hazard regression analysis and were determined to have good prognostic/recurrence predictive value. The diagnostic model constructed with these four angiogenic genes was also confirmed to accurately distinguish between HCC and normal/proliferative nodules. These four angiogenic genes are highly expressed in HCC tissues and work synergistically.

EGF is a growth factor that plays an important role in cell growth, proliferation and migration by binding to its receptor EGFR [32]. Previous immunohistochemistry data have confirmed that EGF is highly expressed in many human malignancies, such as breast cancer and ovarian cancer [33]. Animal model studies have suggested that targeted overexpression of epidermal growth factor (EGF) induces the formation of highly malignant HCC in mice, and its receptor EGFR is also overexpressed in HCC tissues [34]. Given that angiogenesis plays an important role in the development of HCC and that EGFR has been shown to be an angiogenesis promoting factor in previous studies [35], exploring the molecular effects of EGF/EGFR on HCC cells through angiogenesis can better characterize the progression of HCC and identify potential therapeutic targets. Matrix metalloproteinases (MMPs) are a class of extracellular matrix-degrading proteases that affect angiogenesis, wound healing and other physiological and pathological processes, and they are key drivers of tumor progression [36]. MMP3, a member of the MMP family, has been shown to be highly expressed in pancreatic ductal adenocarcinoma (PDA), squamous cell carcinoma (SCC), and breast cancer [37–39], and there is evidence that MMP3 overexpression increases vascular density [40]; however, the existence of molecular links between MMP3 and HCC has not been specifically reported. AURKB is a member of the Aurora kinase B (AURKB) Aurora kinase subfamily, encoding a serine/threonine kinase, and it was determined that aberrant expression of AURKB is associated with tumorigenesis and progression [41]. AURKB has been shown to be highly expressed in thyroid cancer [42] and has value as a potential target marker for renal cell carcinoma [43]. ENFA3 can be expressed in a variety of tissue cells, and the expression of ENFA3 is also detected in various human cancers. For example, ENFA3 is upregulated in liver cancer and colon cancer and downregulated in kidney cancer [44].

Given the complexity of the mechanism of tumor carcinogenesis, fighting cancer has been a long and arduous task for many years [45–47]. In the field of anti-cancer, especially for HCC and other types of cancers that are prone to appear due to changes in various chemical substances, inflammations and molecular markers, the carcinogenic mechanism and corresponding drug development have made gratifying progress [48–50]. In this study, we also aimed to explore the practical value of these angiogenic genes in clinically targeted therapy, we found through pharmacological analysis that piperlongumine was an inhibitor of EGF/EGFR. Piperlongumine is an alkaloid isolated from the long pepper fruit, and its pharmacological properties have been extensively studied in cancer treatment. The antitumor activity of piperlongumine has been revealed through its cytotoxicity toward a variety of cancer cell lines, such as gastric cancer, lung cancer, colon cancer and cholangiocarcinoma, and in several animal cancer models [51, 52]. The anti-tumor effect of piperlongumine is achieved through inhibiting cell proliferation [53], promoting apoptosis [54] and inhibiting cell migration [55]. However, the anti-tumor effect of piperlongumine on HCC and its mechanism have not been specifically studied, and the effect of piperlongumine on angiogenesis and its pathways are still unclear. In this study, we performed in vitro cell experiments to confirm that piperlongumine did have an anti-tumor effect on HCC, which was achieved through the inhibition of EGF/EGFR signaling pathway-induced angiogenesis. Thus, our findings also have applicability in clinical practice, which could provide new choices for the treatment and management of HCC patients.

Inevitably, our current research does have some limitations. Firstly, this study used the LASSO method to screen for angiogenic genes related to the prognosis of HCC. In the process of adjusting the regression coefficients to filter the index weights, some important elements that contribute similarly may be ignored. Secondly, the independent verification of the prognosis and recurrence models and the establishment of the nomogram are mainly based on RNA sequencing data and corresponding clinical information from samples in the public data sets TCGA and ICGC. The main considerations are traditionally recognized clinical factors that have a significant impact on the carcinogenicity of HCC, some important clinical factors with similar contributions may be missed, such as endogenous or exogenous chemicals, lifestyle, eating style, and so on, and the difference in sample follow-up time will also affect the scientific prediction of the models. In addition, the study has not been prospectively tested in clinical trials. The predictive performance of prognostic and diagnostic features associated with angiogenesis and corresponding nomograms needs to be further validated in subsequent multicenter clinical trials and prospective studies. While the data used as the validation set of the prognosis model and the recurrence model were obtained from the GEO database, the sample size contained was not large enough, and it also has an impact on the specific verification of predictive performance. The potential mechanism of angiogenesis genes in the prognosis and recurrence of liver cancer remains to be explored.

## CONCLUSIONS

In summary, we established and validated diagnostic, prognostic, and recurrence models of four angiogenic genes with good diagnostic and predictive value for HCC. The expression characteristics of these four angiogenic genes were significantly associated with the carcinogenicity and clinical outcome of HCC. Pharmacological analysis of the inhibitor of EGF/EGFR, piperlongumine, had been shown to have an anti-tumor effect on HCC, which was achieved through inhibiting angiogenesis via the EGF/EGFR signaling pathway. Our findings will provide clinicians with new and effective ideas for the treatment and management of HCC in clinical practice. Additionally, these data can also provide new application ideas for other kinds of tumors.

## MATERIALS AND METHODS

### Angiogenic genes

A total of 132 angiogenesis-related genes were systematically obtained from relevant studies in the Pubmed, EMBASE, Google Scholar, Web of Science and The Cochrane Library by using the search terms: “angiogenesis” OR “angiogenic”. The relevant public papers were downloaded as of October 2019. The references were referred to additional information.

### Acquisition of mRNA sequencing data associated with HCC

The mRNA-sequencing data and corresponding clinical information associated with HCC patients were downloaded from TCGA and ICGC databases. There were 370 HCC tissue samples and 50 normal tissue samples as of July 8, 2019, for a total of 19677 encoding mRNA sequences in TCGA HCC cohort, and 202 normal samples, 243 HCC samples and 22913 mRNA sequences in the ICGC HCC cohort. Since these mRNA-sequencing data were obtained from TCGA and ICGC databases and were freely available to the public, and as this study also strictly followed the publication guidelines and access policies of these databases, this study does not require ethical review and approval from an Ethics Committee.

### Identification of differentially expressed genes (DEGs) between HCC and adjacent noncancerous tissues

A total of 132 angiogenic genes were matched with HCC-associated mRNA obtained from the RNA-sequencing platform in TCGA database and the ICGC database to identify angiogenic genes associated with HCC. Then, we determined the differentially expressed genes (DEGs) by limma, an R package, with an absolute log2-fold change (FC) > 1 and an adjusted P value < 0.05.

### Definition of an angiogenesis-related prognostic signature

The expression profiles of differentially expressed angiogenic genes were assessed via LASSO, univariate and multivariate Cox regression analysis to evaluate the prognostic value of these genes for OS in HCC patients. We first implemented the least absolute shrinkage and selection operator (LASSO) method with an L1 penalty, which reduces the regression coefficient by applying a penalty proportional to its size, so that the number of indicators with a final weight of nonzero is relatively small and so that most potential indicators are reduced to zero [56], thereby reducing the number of angiogenic genes and screening for more relevant angiogenic genes. In the LASSO-penalized Cox regression analysis, we performed 1000 substitution samplings in the data set and selected marker genes with a recurrence frequency greater than 900. Subsequently, we adopted univariate and multivariate Cox regression analysis for these genes to determine key genes with the best prognostic performance. A P value for a gene <0.01 in analyses was considered to represent statistical significance, and the gene was viewed as a crucial prognostic factor in prognostic prediction. Then multivariate Cox regression analysis integrated these statistically significant genes, and the genes univariate Cox regression found no statistical significance but significant clinically relevant, and selected variables by forward selection and backward elimination. The optimal set of genes was obtained according to the test level of the selected variable> the test level of the excluded variable. Finally, we integrated these key genes to establish an optimal apoptosis-related prognostic signature.

### Validation analysis of the prognostic signature

Using a linear combination of regression coefficient (β) in a multivariate Cox regression model and angiogenic gene expression levels, we determined a prognostic risk score based on the angiogenesis-related prognostic signature. Prognostic index (PI) = (β* expression level of EGF) + (β* expression level of MMP3) + (β* expression level of AURKB) + (β* expression level of EFNA3). Using X-tile software, we confirmed the optimal cut-off value and thus divided 370 HCC patients into high-risk and low-risk groups. To assess the predictive performance of this angiogenesis-related prognostic signature, we established a Kaplan-Meier (K-M) survival curve and a time-dependent receptor working characteristic (ROC) curve.

### Independence of the prognostic signature from other clinical variables

To assess the independence of this angiogenesis-related prognostic signature in predicting OS in HCC patients compared to other clinical features (age, gender, weight, AFP, vascular tumor cell infiltration, clinical stage and pathological grading), we performed univariate and multivariate Cox regression analysis on these variables. A bilateral P value <0.05 was considered statistically significant. The hazard ratio (HR) and 95% confidence interval (CI) for each variable were calculated.

### Construction and validation of a nomogram

Based on the multivariate Cox regression model, we determined independent prognostic factors associated with OS in HCC patients with HCC (P < 0.05) and used rms R software to integrate these independent prognostic factors and construct a predictive nomogram and a corresponding calibration map. The calibration map is validated by calibration and discrimination. In this study, we graphically plotted the probability of collinear prediction to the observed rate to evaluate the alignment curve of the nomogram. The closer the calibration curve was to the reference line, representing the best predictor (at the 45° line), the better the prognostic performance of this nomogram. We also adopted a consistency index (C index), which was calculated using a bootstrap method with 1000 resamples to validate the predictive accuracy between the nomogram and the actual result. The C index ranged from 0.5 to 1.0, which means that the ability to correctly distinguish between results and models ranged from random to perfect. ROC curve analyses were performed to validate and compare the predictive performance between single independent prognostic factors and the nomogram. Meanwhile, the, decision curve analysis (DCA) was performed to evaluate the clinical benefit of the nomogram compared to a single prognostic factor. DCA can quantify the clinical applicability of a nomogram by analyzing the clinical outcomes of nomogram-based decisions, and it has great value in determining alternative diagnostic and prognostic strategies [57]. A P value <0.05 was considered to represent statistical significance.

### Internal and external validation of gene expression levels of HCC-associated angiogenic genes

Further internal and external validation of the expression patterns of the four angiogenic genes were performed in the cohort from TCGA and the ICGC cohort. Using Wilcoxon signed rank tests in Prism 7.0 (GraphPad, San Diego, CA, USA), we compared the expression profiles of the four angiogenic genes between HCC and normal tissues. A bilateral P value <0.05 was considered to represent statistical significance. In the Human Protein Atlas database, we identified the expression patterns of the four genes using images of HCC and normal tissues. We also explored the extractions among these genes. Finally, the predictive value of each gene for OS in HCC patients was validated using ROC curve analysis.

### Cell culture

Human HCC cell lines (SK-HEP1 and SMMC-7721) were purchased from American Type Culture Collection (ATCC) (Manassas, VA, USA). The cell lines were cultured in DMEM (Gibco, Eggenstein, Germany) supplemented with 10% heat-inactivated fetal bovine serum (Gibco, Eggenstein, Germany), and the cell lines were grown in a humidified cell incubator with an atmosphere of 5% CO^2^ at 37°C.

### Cell counting kit-8 (CCK8) assay

SK-HEP1 and SMMC-7721 cells (3000 cells/well) were plated in 96-well plates (n=5/group) and were incubated in a humidified cell incubator with an atmosphere of 5% CO2 at 37°C for 24 hours. Cells were treated with piperlongumine (0-10 μmol/ml) for 24, 48 and 72 hours, and CCK8 reagent was added to the wells, and the cells were incubated for an additional 2 hours. The OD was then measured at 450 nm using a microplate reader.

### 5-ethynyl-2´-deoxyuridine (EdU) cell proliferation assay

SK-HEP1 and SMMC-7721 cells (3000 cells/well) were plated and incubated in complete medium in a 96-well plate (n=5/group) with an atmosphere of 5% CO2 at 37°C overnight. Then, the cells were treated with piperlongumine (0-8 μg/ml) or EGF (200 ng/ml) and incubated with 50 μM EdU for 6 hours. SK-HEP1 and SMMC-7721 cells were then fixed, permeabilized and EdU stained according to the manufacturer's protocol. Proliferative cells were labeled with a Click-iTTM EdU imaging kit (Ruibo, Guangzhou, China). Cell nuclei were stained with Hoechst 33342 (1 μg/ml) for 10 minutes. The proportion of cells that incorporated EdU was detected by fluorescence microscopy.

### In vitro HUVEC tube formation assay

Culture plates (96-well) were coated with ECMatrix^TM^ according to the manufacturer’s instructions for 2 hours. HUVECs in endothelial cell growth medium (EGM, CC-3162, Lonza) treated with piperlongumine solutions (2 μmol/ml) were added to the precoated plate at a density of 1×10^4^ cells/ml. The cultures were incubated in a 37°C humidified incubator for 12 hours, and then an inverted light microscope was used to photograph in vitro angiogenesis at the center of the well.

### Western blot analysis

SK-HEP1 and SMMC-7721 cells were treated with an inhibitor and lysed with protein lysis buffer. Then, the debris was removed by centrifugation at 12,000 rpm for 10 min at 4°C. Protein samples were separated using 10% gels via sodium dodecyl sulfate-polyacrylamide gel electrophoresis (SDS-PAGE) and were then transferred to PVDF membranes. Freshly prepared 5% nonfat milk in TBST was used to block the blots for 1.5 h at room temperature on a shaker. The blots were incubated overnight with a specific primary antibody on a shaker at 4°C to probe the blot, and this was followed by incubation with a horseradish peroxidase-conjugated secondary antibody and treatment with an ECL kit (Bio-Rad) for protein detection. The antibodies used in the study were list in supporting information.

### Quantitative real-time PCR (qPCR)

Total RNA was extracted from cultured SK-HEP1 cells, SMMC-7721 cells and HUVECs treated with piperlongumine using TRIzol reagent (Invitrogen, Carlsbad, USA) according to the manufacturer’s instructions. Then, total RNA was reverse transcribed using a cDNA reverse transcription kit (TransGen, Guangzhou, China), and the obtained cDNA was amplified using a SYBR Green PCR kit (TransGen, Guangzhou, China). Quantitative real-time PCR analysis was performed to detect expression in samples. Each experiment was repeated at least three times. The primers used for qRT-PCR were obtained from RiboBio Co., Ltd. The expression of genes was analyzed using 2-ΔΔCT methodology. The specific content of the relevant primers used in qPCR can be found in the support information.

### Statistical analysis

All data are presented as the mean ± standard deviation (SD), and each data point represents at least five samples. The differences between two groups were calculated using Student’s two-sided t-tests. P<0.05 was considered to represent a statistical significance.

## Supplementary Material

Supplementary Figures
