# Supplementary material for: Identification and validation of the angiogenic genes for constructing diagnostic, prognostic, and recurrence models for hepatocellular carcinoma
Source: Aging (Albany NY). 2020 May 6;12(9):7848–73. doi: 10.18632/aging.103107 (PMC7244068; doi:10.18632/aging.103107)
Supplement: Supplementary Figures [file aging-12-103107-s001..pdf]

SUPPLEMENTARY FIGURES

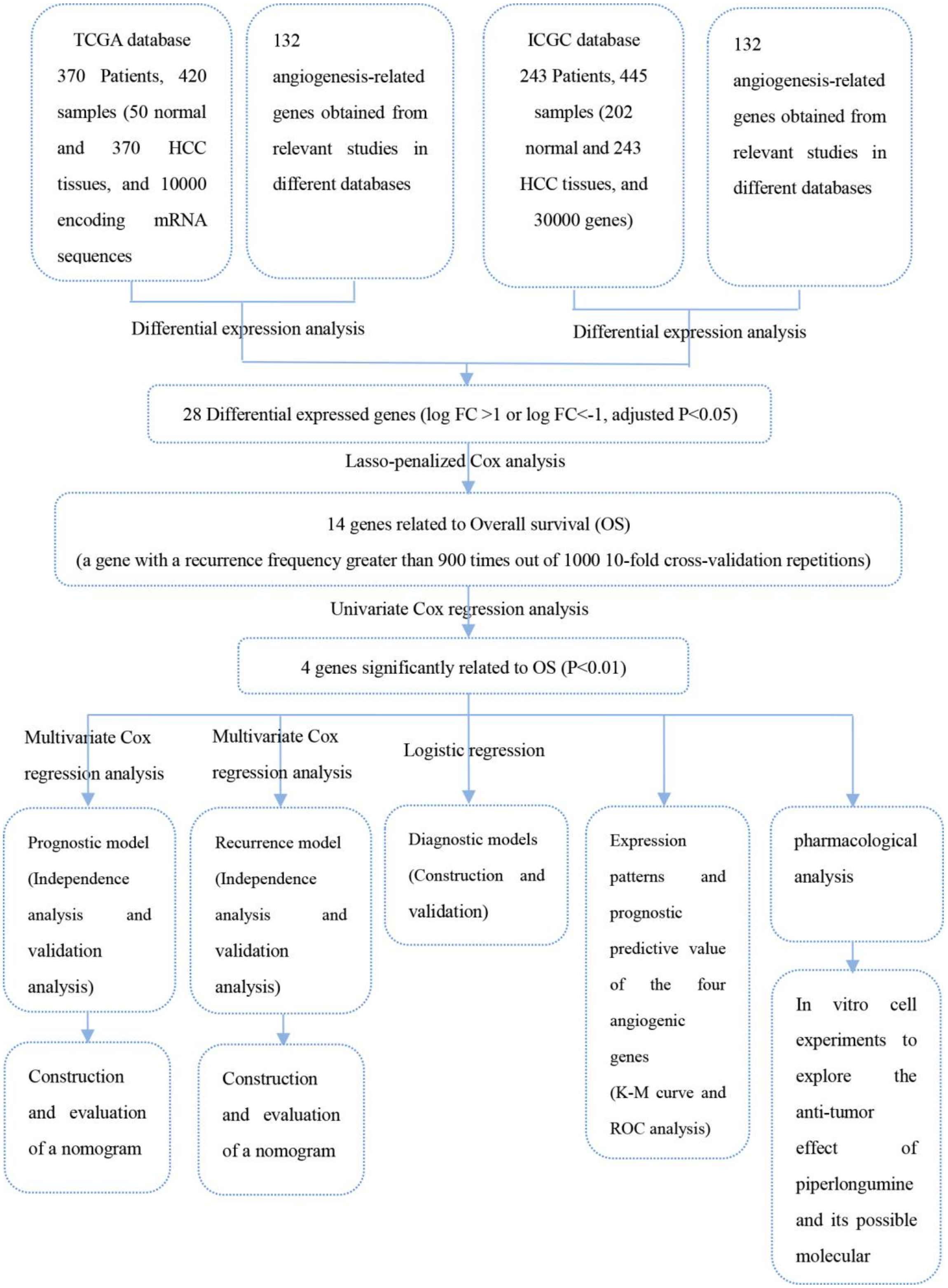

Supplementary Figure 1. An overall flow chart explaining to indentify the angiogenic genes for constructing diagnostic, prognostic, and recurrence models in hepatocellular carcinoma.

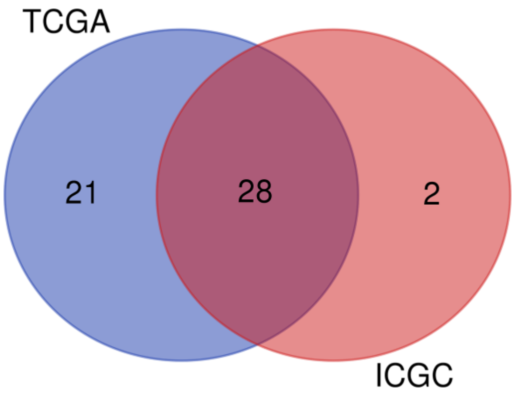

Supplementary Figure 2. Commonly differentially expressed angiogenic genes in TCGA and ICGC databases.

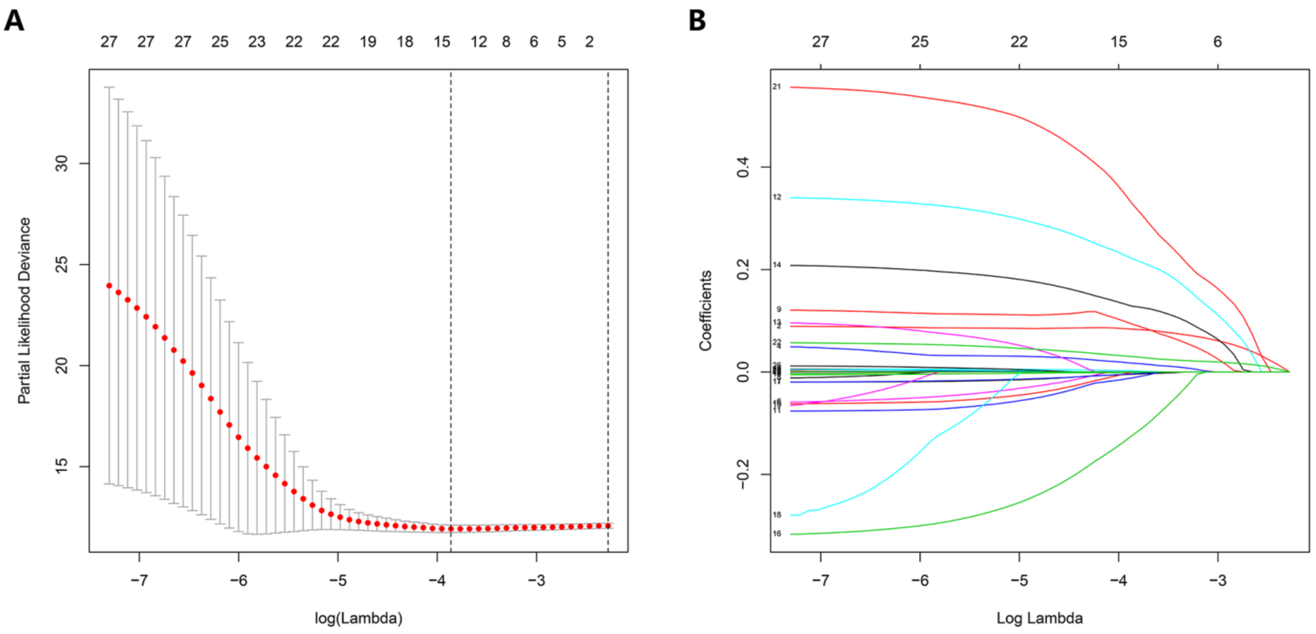

Supplementary Figure 3. Identification of key angiogenic genes closely associated with prognosis of HCC. LASSO Cox regression (**A**, **B**) were performed to identify the angiogenic genes closely related to OS of HCC.

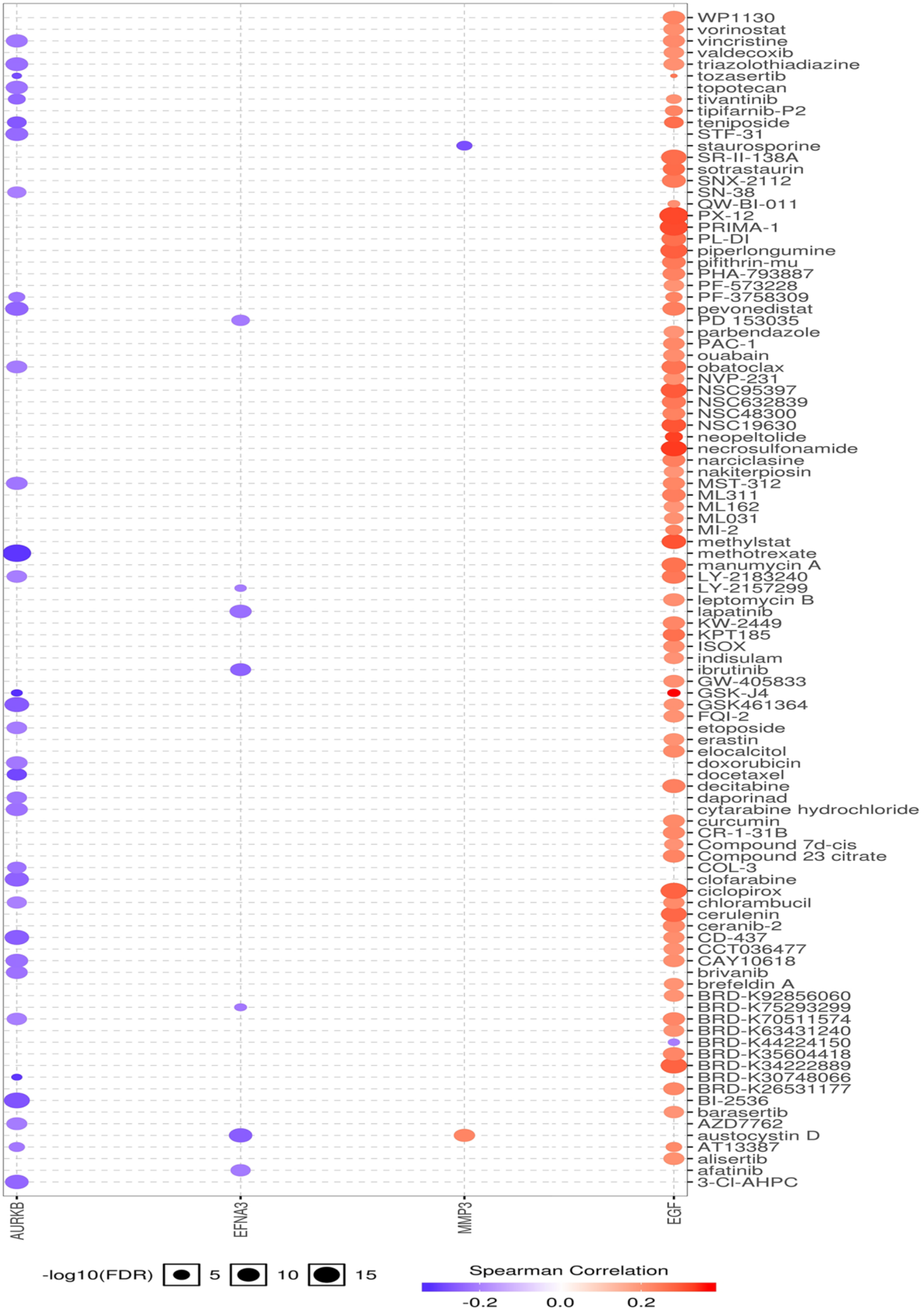

Supplementary Figure 4. Inhibitors of the angiogenic genes. Data was obtained from the Cancer Therapeutics Response Portal (CTRP) database (http://portals.broadinstitute.org/ctrp/).

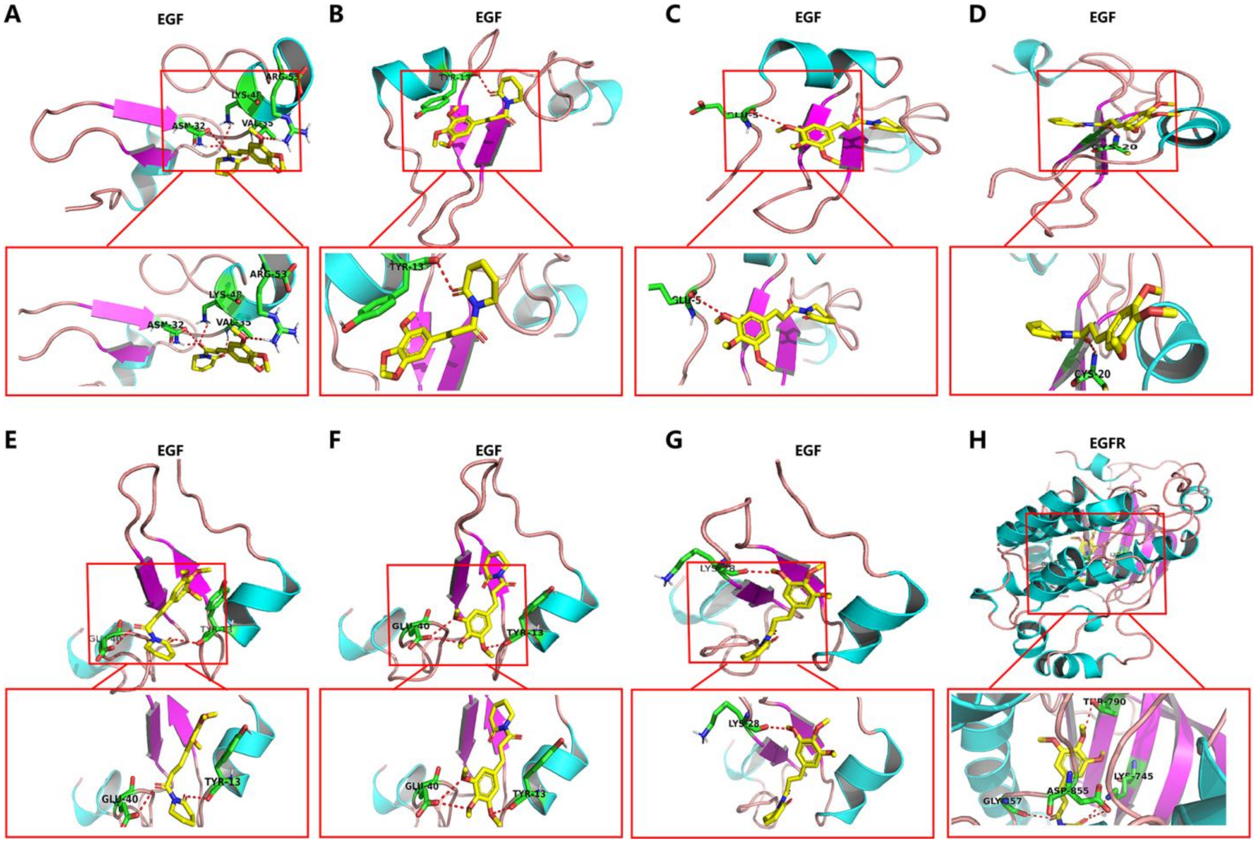

Supplementary Figure 5. Molecular docking for targeted drug identification. Specific binding sites of piperlongumine to EGF (**A**–**G**) and EGFR (**H**).
